# Narrow-Band Ultraviolet B Phototherapy Ameliorates Acute Graft-Versus-Host Disease of the Intestine by Expansion of Regulatory T Cells

**DOI:** 10.1371/journal.pone.0152823

**Published:** 2016-03-31

**Authors:** Akari Hashimoto, Tsutomu Sato, Satoshi Iyama, Masahiro Yoshida, Soushi Ibata, Ayumi Tatekoshi, Yusuke Kamihara, Hiroto Horiguchi, Kazuyuki Murase, Yutaka Kawano, Kohichi Takada, Koji Miyanishi, Masayoshi Kobune, Shingo Ichimiya, Junji Kato

**Affiliations:** 1 Department of Medical Oncology and Hematology, Sapporo Medical University School of Medicine, Sapporo, Japan; 2 Department of Human Immunology, Research Institute for Frontier Medicine, Sapporo Medical University School of Medicine, Sapporo, Japan; University of Alabama at Birmingham, UNITED STATES

## Abstract

Narrowband ultraviolet B (NB-UVB) has been widely used in dermatological phototherapy. As for the application of NB-UVB phototherapy to graft-versus-host disease (GVHD), we previously reported that it was highly efficacious for cutaneous lesions of acute GVHD (aGVHD) and that expansion of regulatory T (Treg) cells induced by NB-UVB might be one of the mechanisms. In order to examine whether NB-UVB irradiation through expansion of Treg cells is effective for the treatment of not only cutaneous aGVHD but also aGVHD of inner organs such as the intestine or liver, we conducted experiments in which a murine lethal aGVHD model, characterized by severe involvement of the intestine, was irradiated with NB-UVB. We found that NB-UVB irradiation improved the clinical score and survival rate. The pathological score of aGVHD was improved in all affected organs: intestine, liver, and skin. In the serum of mice irradiated with NB-UVB, the levels of Treg cells-associated cytokines such as transforming growth factor beta (TGFβ) and interleukin-10 (IL-10) were elevated. The numbers of infiltrating Treg cells in inflamed tissue of the intestine and those in spleen were increased in mice treated with NB-UVB. This is the first report demonstrating that NB-UVB phototherapy has the ability to ameliorate intestinal aGVHD through the expansion of Treg cells.

## Introduction

Allogeneic hematopoietic stem cell transplantation (allo-HSCT) is an effective treatment for various malignant and nonmalignant hematologic disorders. Nevertheless, graft-versus-host disease (GVHD) remains a life-threatening complication of allo-HSCT [1]. Acute GVHD (aGVHD), a leading cause of nonrelapse mortality after allo-HSCT, is characterized by dysfunction in three organ systems: the skin, liver, and gastrointestinal (GI) tract [2].

Corticosteroids are the cornerstone of initial therapy for aGVHD, leading to complete response in 25%-69% of patients [3–7]. Patients not responding to steroids have a dismal prognosis, with poor survival [3,8]. Numerous agents for treating steroid-refractory aGVHD have been studied [8,9]; however, to date, there is no consensus for the treatment of refractory aGVHD [10].

For patients with skin aGVHD refractory to standard corticosteroid treatment with no gut/liver involvement, we and others have previously reported that narrow-band ultraviolet B (NB-UVB) phototherapy was a highly effective, safe and well tolerable treatment choice [11–16]. NB-UVB is a light with a peak at around 311 nm emitted by fluorescent bulbs (Philips model TL-01), which exclude shorter, photobiologically more erythrogenic wavelengths in UVB [12,17]. The introduction of NB-UVB is an advance in UVB-based phototherapy and currently it is one of the most commonly used phototherapy devices. In our previous report, eight out of eleven patients with refractory skin aGVHD achieved an objective complete response with NB-UVB irradiation [11].

Concerning the mechanisms whereby NB-UVB mediates such an excellent therapeutic effect on skin aGVHD, we demonstrated that not only putative direct effects on inflammatory cells in the skin but also indirect effects on systemic immunity by the induction of regulatory T (Treg) cells [11] were involved.

Treg cells are a subset of T cells defined by coexpression of CD4, CD25 and transcription factor Forkhead box protein 3 (FoxP3). These cells account for 5–10% of circulating CD4^+^ T cells, suppress autoreactive lymphocytes, and control innate and adaptive immune responses [18]. It has been shown that Treg cells are capable of reducing the severity of GVHD [19,20]. Rapamycin plus interleukin-2 (IL-2) treatment resulted in improved survival and a reduction of aGVHD lethality in a mouse model, associated with an increased expansion of donor-type Treg cells [19]. Further, low-dose IL-2 was safely administered to patients with chronic GVHD, reducing the severity of GVHD in parallel with expansion of Treg cells [20].

To the best of our knowledge, our previous report [11] was the first report demonstrating the possibility that NB-UVB irradiation increased Treg cells in skin aGVHD patients. If so, NB-UVB irradiation should be able to ameliorate not only skin aGVHD but also aGVHD of internal organs such as the GI tract and liver. To address this issue, we conducted experiments in which a lethal mice model of aGVHD with GI tract involvement [21–24] was treated with NB-UVB irradiation. The results obtained in this study suggest that the indication of NB-UVB phototherapy could be expanded to aGVHD of internal organs.

## Materials and Methods

### Mice

BALB/c (H-2K^d^) and C57BL/6 (H-2K^b^) female mice of 6–7 weeks of age and weighing 19–21 g were obtained from Charles River Laboratories (Yokohama, Japan). The mice were kept under specific pathogen-free conditions with a 12 hour light: 12 hour dark cycle and free access to food and water, and received humane care in compliance with Institutional Guidelines. All experiments were approved by the Animal Care and Use Committee of Sapporo Medical University.

### Bone marrow transplantation

Allogeneic (C57BL/6 to BALB/c) transplanted animals were generated as previously described [21,23–25]. Recipient BALB/c mice received a lethal dose of irradiation (4 Gy, twice a day) at day 0. On the same day, C57BL/6 donors were sacrificed. Bone marrow cells and splenocytes were extracted from femurs and whole spleens, respectively, from which single cell suspensions were prepared in RPMI 1640 (Gibco BRL, Tokyo, Japan). Recipient BALB/c mice then received 5×10^6^ bone marrow cells and 5×10^5^ splenocytes via tail vein injection and were monitored for weight and condition three times a week. The clinical severity of aGVHD was evaluated according to an assessment score as described previously [26].

### NB-UVB irradiation

Two PL-L 36W/01 fluorescent lamps (Philips, Eindhoven, Holland), which emitted light in a narrow peak at around 311 nm exclusively, were used to irradiate the mice with NB-UVB. The irradiation equipment was built by YAYOI CO., LTD (Tokyo, Japan). The irradiation intensity was 2.0 mW/cm^2^ at a distance of 25 cm, as measured by Variocontrol (Waldmann, Villingen-Schwenningen, Germany). To obtain an irradiation dose of 0.3 J/cm^2^, which was the approximate sub-minimum erythema dose (MED) for albino hairless mice [22], the irradiation time of 150 sec was calculated by the formula: Irradiation time (sec) = Irradiation dose (J/cm^2^)×1,000/Light intensity (mW/cm^2^). The mice were irradiated after shaving their backs starting on day 13 after BMT, just before the start of bloody diarrhea, until day 70. The frequency of irradiation was three times weekly since Dawe et al.[27] found no significant difference in clearing rates of psoriasis in human patients between five times weekly versus three times weekly.

### Histopathology

Representative samples of intestine, lung, skin, and liver were obtained from transplanted recipients at day 35 after BMT and fixed in 10% neutral-buffered formalin. The euthanasia method used for sacrifice of mice was intraperitoneal injection of at least 200 mg/kg sodium pentobarbital. Samples were then embedded in paraffin, cut into 5-μm-thick sections and stained with hematoxylin and eosin. Images were visualized with an Eclipse Ni-U microscope and a Plan APO 20×/0.75 lens (Nikon, Tokyo, Japan). Image acquisition was performed with a DS-Ri1 camera and NIS-Elements Documentation version 4.20 software (Nikon). A semiquantitative scoring system was used to account for histologic changes as previously described [28].

### Immunohistochemistry

Expression of CD3 and FoxP3 in sections of intestine, lung, and skin was assessed by immunohistochemistry using the standard protocol. In brief, after deparaffinization and rehydration, tissue sections were incubated with rat monoclonal anti-mouse T-cell marker CD3e (TCRE), clone HH3E (DIA-303) (Dianova GmbH, Hamburg, Germany), or with rat monoclonal anti-mouse/rat Foxp3, clone FJK-16s (14–5773) (eBioscience, San Diego, CA), overnight at 4°C. The immune complex of CD3/anti-CD3 antibody or FoxP3/anti-FoxP3 antibody on the tissue section was detected using anti-rat immunoglobulin G antibody conjugated with biotin, which was visualized with the 3,3’-diaminobenzidine peroxidase substrate kit (Vector Labs, Burlington, ON). FoxP3^+^ cells were semiquantitatively counted under ×400 magnification [high-power field (hpf)] in a blinded fashion. At least ten non-overlapping views were obtained for each section.

### Serum cytokine analysis

Serum was obtained from surviving recipient mice on day 35 after BMT and stored at 80°C. Levels of interferon gamma (IFNγ), IL-2, IL-4, IL-17, transforming growth factor beta (TGFβ), and IL-10 were simultaneously determined by the enzyme-linked immunosorbent assay (ELISA) method using the Quantikine ELISA kits (R&D Systems, Minneapolis, MN) according to the instructions provided by the manufacturer. A total of five samples were analyzed in each group.

### Flow cytometric analysis

Splenocytes were obtained from mice at day 35 after BMT. To detect Treg cells, the Treg Detection Kit (CD4/CD25/FoxP3) mouse (Miltenyi Biotec, Bergisch Gladbach, Germany) was used according to the instructions provided by the manufacturer. Briefly, the surface of splenocytes was first stained with monoclonal anti-CD4 antibodies (clone GK1.5) conjugated to fluorescein-isothiocyanate (FITC) and monoclonal anti-CD25 antibodies (clone 7D4) conjugated to R-phycoerythrin (PE). After incubation with Fixation/Permeabilization solution, splenocytes were stained intracellularly with monoclonal anti-FoxP3 antibodies (clone 3G3) conjugated to allophycocyanin (APC). Then, the stained splenocytes were analyzed on the BD FACSCanto II (BD Biosciences, San Jose, CA) with FlowJo software 7.6.1 (Treestar, Ashland, OR).

### Statistical analysis

All statistical analyses were performed using GraphPad Prism version 5.0 (GraphPad Software, La Jolla, CA). All values are presented as mean±standard error of the mean (SEM). Statistical significance was determined using Student’s t-test in the case of normally distributed data, otherwise the Mann–Whitney U test. For comparisons of data from the same mouse, the paired Student’s t-test was used. To analyze the statistical significance of differences in survival curves constructed using the Kaplan-Meier method, log-rank test was used. Statistical significance was defined as p<0.05.

## Results

### NB-UVB irradiation prolonged the survival of aGVHD mice

**Fig 1:**
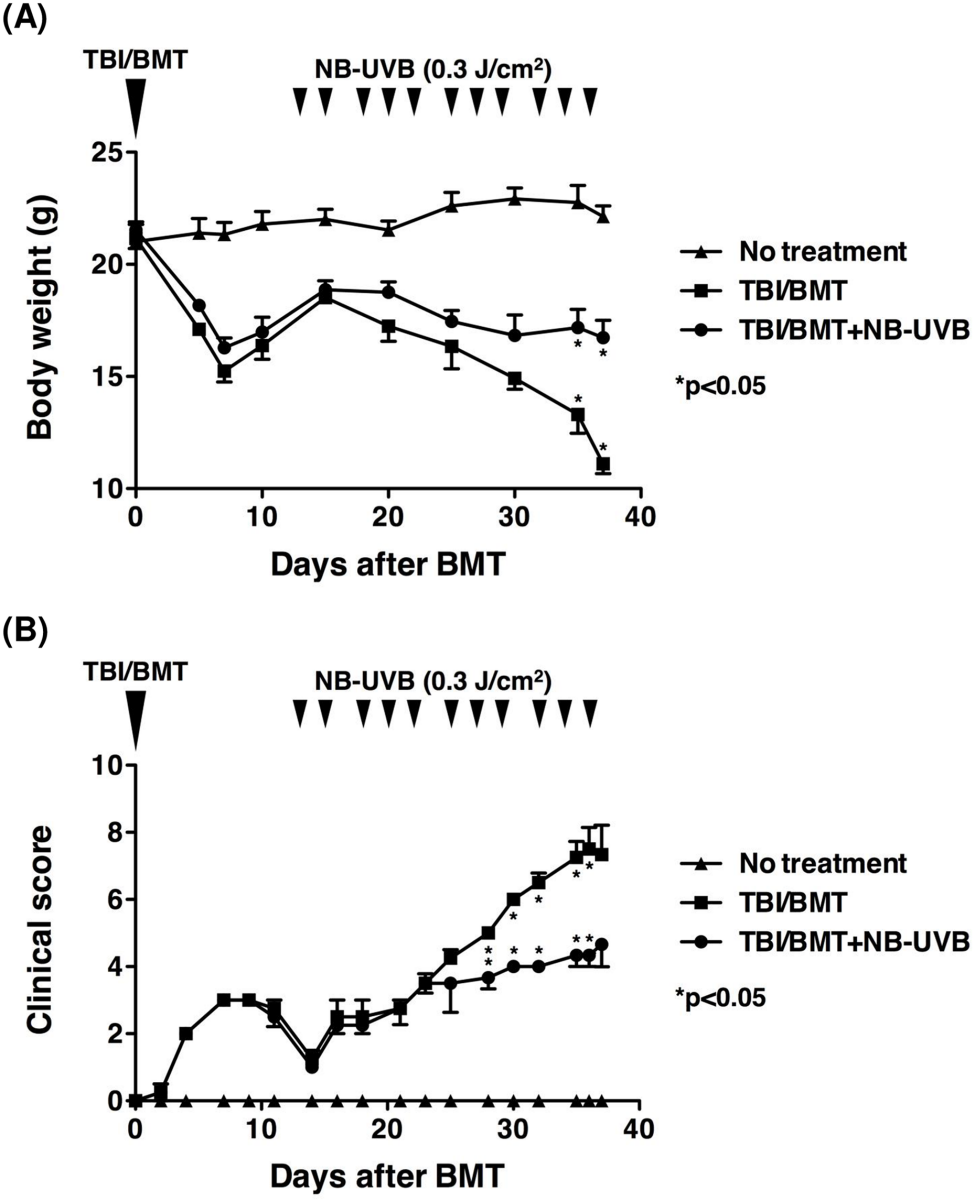
Effects of NB-UVB irradiation on body weight and clinical score of aGVHD mice. Recipient mice were given TBI followed by BMT with spleen cells from donor mice on day 0 (“TBI/BMT” group). After undergoing TBI/BMT, mice were irradiated with NB-UVB (“TBI/BMT+NB-UVB” group). The mice without TBI/BMT were the “No treatment” group. All mice were weighed (A) and their clinical score was calculated as described in the “Materials and Methods” (B). The data are representative of three separate experiments and presented as the mean±SEM.

Mice in the “TBI/BMT” group underwent total body irradiation (TBI) followed by bone marrow transplantation (BMT) on day 0. As shown in Fig 1A, the body weight of the mice decreased transiently until day 7 and this may have been due to side effects of TBI. Then, their body weight increased until day 15 and it decreased again after day 15 with worsening bloody diarrhea. A second group of mice treated with TBI/BMT was irradiated with NB-UVB three times a week starting on day 13, just before the start of bloody diarrhea (“TBI/BMT+NB-UVB” group). The mice in the “TBI/BMT+NB-UVB” group had no bloody diarrhea and maintained a higher body weight than that in the “TBI/BMT” group with statistical significance at day 35 (p = 0.0088) and day 37 (p = 0.0019).

In the same experimental setting mentioned above, the clinical severity of aGVHD was evaluated according to the assessment score [26], which includes five parameters: weight loss, posture (hunching), activity, fur texture, and skin integrity. Higher scores indicate more severe clinical symptoms. In the “TBI/BMT” group, the clinical score increased until day 7 and then decreased until day 14; after day 14, the clinical score showed a steady increase (Fig 1B). The rise in clinical score after day 23 in the “TBI/BMT+NB-UVB” group was less sharp than that in the “TBI/BMT” group. The score of the “TBI/BMT+NB-UVB” group was lower than that of the “TBI/BMT” group with statistical significance at day 28 (p = 0.0049), day 30 (p<0.0001), day 32 (p = 0.0007), day 35 (p = 0.0057), and day 36 (p = 0.0114).

**Fig 2:**
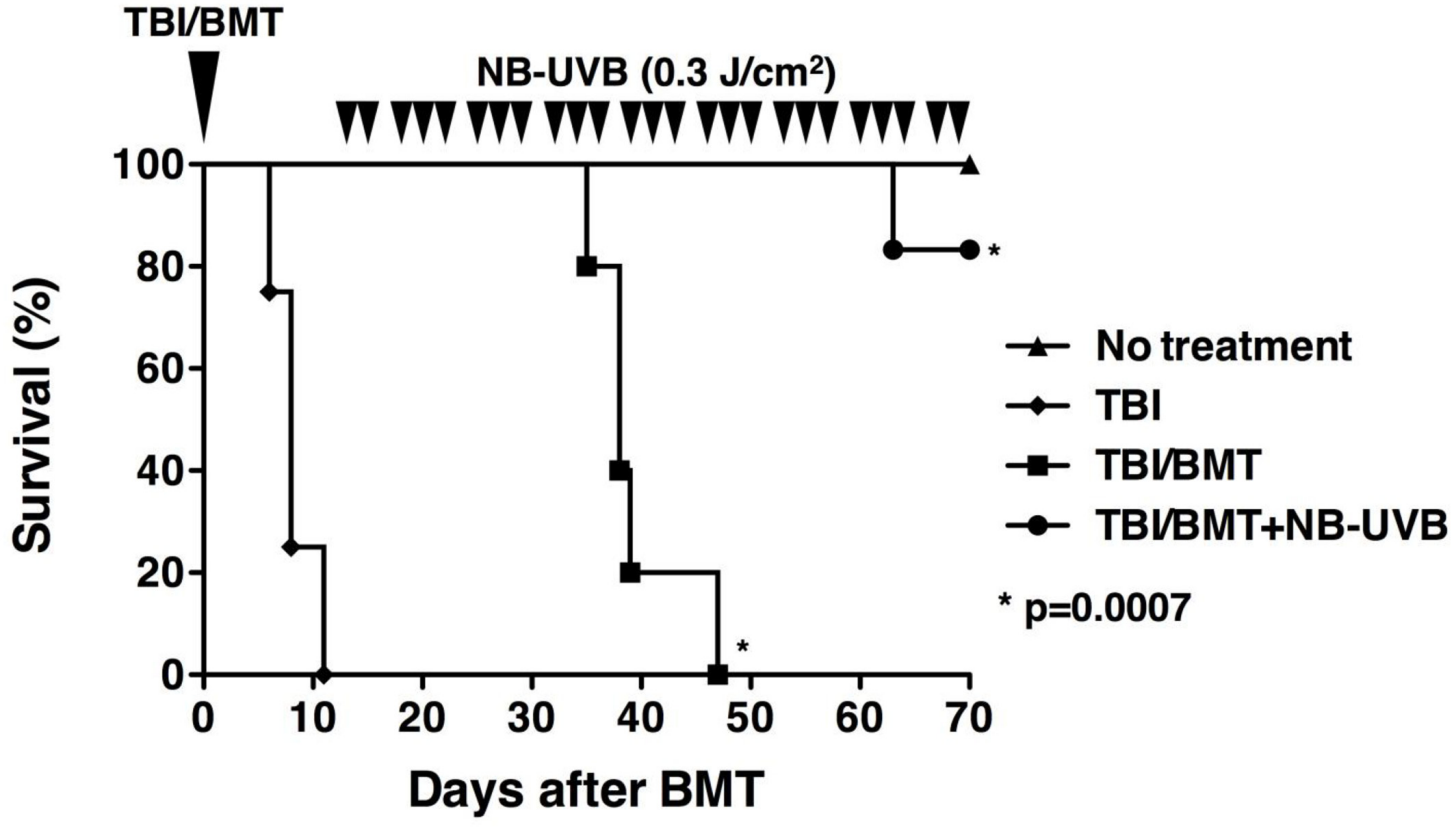
Effect of NB-UVB irradiation on survival of aGVHD mice. The experimental design was the same as that described in the legend of Fig 1. The mice treated with TBI without BMT were the “TBI” group. The data are representative of three separate experiments. The data are representative of three separate experiments and presented as the mean±SEM.

As shown in Fig 2, the mice that underwent TBI at day 0 without BMT (“TBI” group) died between day 6 and 11 with a median survival time (MST) of 8 days. The mice in the “TBI/BMT” group died between day 35 and 47 with a MST of 38 days. On the other hand, only one out of six mice in the “TBI/BMT+NB-UVB” group died on day 63. The other five mice were alive until the end of the observation period on day 70. The survival of the “TBI/BMT+NB-UVB” group was significantly longer than that of the “TBI/BMT” group (p = 0.0007). Of note, the cause of death on day 63 of the mouse in the “TBI/BMT+NB-UVB” group could not be identified in spite of pathological examination; however, findings of severe aGVHD were not observed.

### Pathological findings of aGVHD improved in NB-UVB-irradiated mice

**Fig 3:**
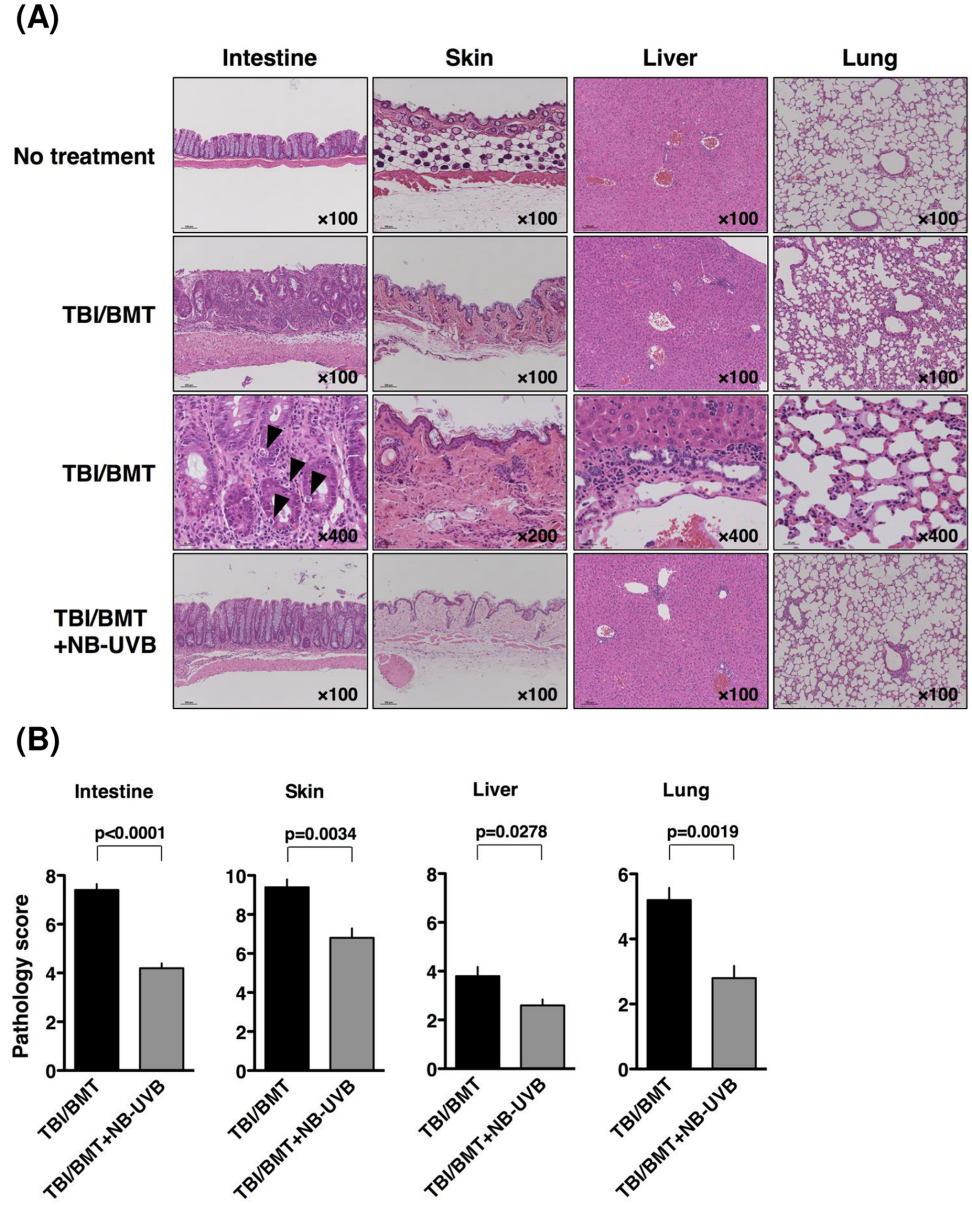
Effects of NB-UVB irradiation on histopathology and pathological score of aGVHD mice. Tissue samples were collected on day 35 after BMT. (A) Photomicrographs in routine hematoxylin and eosin (H&E)-stained sections are presented as a typical image in each group. Magnification is indicated in each photomicrograph. (B) The clinical score was calculated as described in the “Materials and Methods”. The data are representative of three separate experiments and presented as the mean±SEM.

To evaluate the effect of NB-UVB on the histopathology of aGVHD, mice were sacrificed on day 35 after BMT; this day was selected since the mice in the “TBI/BMT” group died between day 35 and 39. Then, tissue sections of target organs (intestine, skin and liver) were prepared. As shown in Fig 3A, the intestine from mice in the “TBI/BMT” group exhibited severe inflammation with ulceration and apoptotic bodies in the majority of crypts, which are characteristics of intestinal aGVHD. Upon NB-UVB treatment, the structural integrity of the villi and crypts of mice was substantially maintained in the “TBI/BMT+NB-UVB” group. In the skin of mice in the “TBI/BMT” group, widespread interface damage, increased collagen density, inflammatory cell infiltration, serous fat atrophy, and loss of hair follicles were observed. With NB-UVB treatment, interface damage and collagen deposition were somewhat modest in the skin of mice in the “TBI/BMT+NB-UVB” group. However, improvement of skin by NB-UVB treatment was not so obvious, perhaps due to direct inflammatory stimulation of NB-UVB to skin. The liver of mice in the “TBI/BMT” group exhibited a few involved tracts with mild infiltration of mononuclear cells. Lymphocyte infiltration was decreased in the liver of mice in the “TBI/BMT+NB-UVB” group.

The pathological findings were evaluated according to the pathological scoring system [28]. NB-UVB irradiation significantly reduced the score of the intestine due to TBI/BMT (7.4±0.2 vs. 4.2±0.2; “TBI/BMT” vs. “TBI/BMT+NB-UVB”; p<0.0001) (Fig 3B). As for skin, NB-UVB reduced the score compared with that in the “TBI/BMT” group not so effectively as in the intestine but with statistical significance (9.4±0.4 vs. 6.8±0.5; “TBI/BMT” vs. “TBI/BMT+NB-UVB”; p = 0.0034). The score of the liver was not very high in the “TBI/BMT” group but the difference between the “TBI/BMT” and “TBI/BMT+NB-UVB” groups was statistically significant (3.8±0.4 vs. 2.6±0.2; “TBI/BMT” vs. “TBI/BMT+NB-UVB”; p = 0.0278).

We further performed histopathological examination of the lungs. Interstitial and alveolar pneumonitis, perivascular lymphocytic inflammation, and lymphocytic bronchiolitis were observed in the “TBI/BMT” group (Fig 3A). These findings were compatible with the clinically defined “idiopathic pneumonia syndrome (IPS)”, encompassing widespread alveolar injury in the absence of infection, cardiac dysfunction, renal failure, and iatrogenic fluid overload, and affecting 5% to 25% of allo-HSCT recipients [29–31]. By the semi-quantitative scoring system of IPS [26], it was clearly demonstrated that NB-UVB irradiation ameliorated IPS with statistical significance (p = 0.0019), as shown in Fig 3B.

### FoxP3^+^ Treg cells increased in NB-UVB-irradiated aGVHD mice

**Fig 4:**
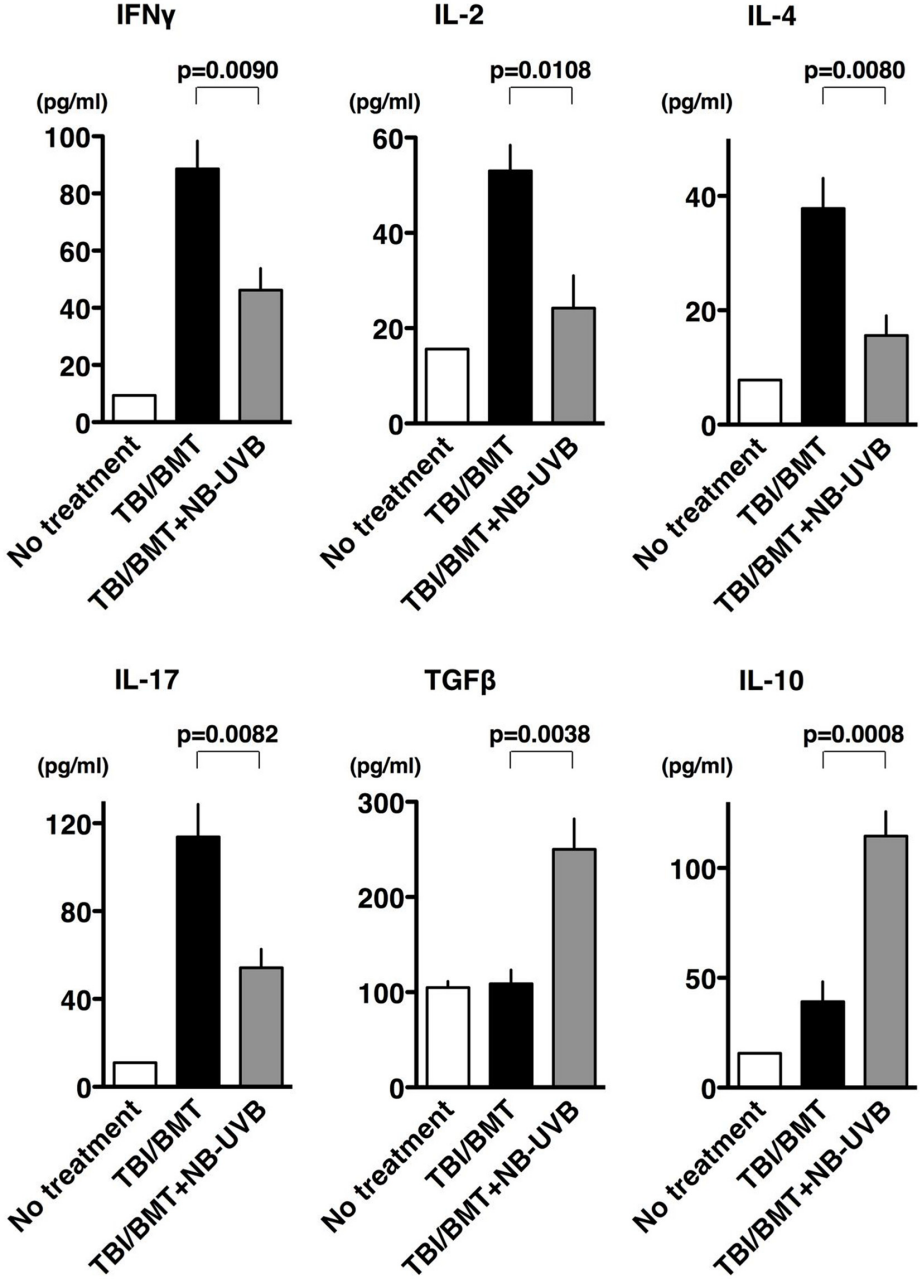
Effects of NB-UVB irradiation on serum cytokines in aGVHD mice. Serum samples were collected on day 35 after BMT. Cytokine levels were determined by ELISA as described in the “Materials and Methods”. The data are representative of three separate experiments and presented as the mean±SEM.

The “cytokine storm” released by massive numbers of alloreactive T cells is considered to be a characteristic event of aGVHD [32]. To determine whether the attenuation of aGVHD in mice irradiated with NB-UVB was a result of its modulating effect on the secretion of various cytokines, the production of IFNγ, IL-2, IL-4, IL-17, TGFβ, and IL-10 was analyzed. The levels of these cytokines in serum of mice on day 35 are shown in Fig 4. TBI/BMT increased the serum levels of cytokines from T helper type 1 (Th1) cells such as IFNγ and IL-2, those from T helper type 2 (Th2) cells like IL-4, and those from T helper type 17 (Th17) cells like IL-17; NB-UVB irradiation significantly reduced the serum levels of these cytokines (p = 0.0090, 0.0108, 0.0080, or 0.0082, respectively). As for the cytokines from Treg cells such as TGFβ and IL-10, the production of these cytokines was strongly upregulated by NB-UVB (p = 0.0038 or 0.0008, respectively).

**Fig 5:**
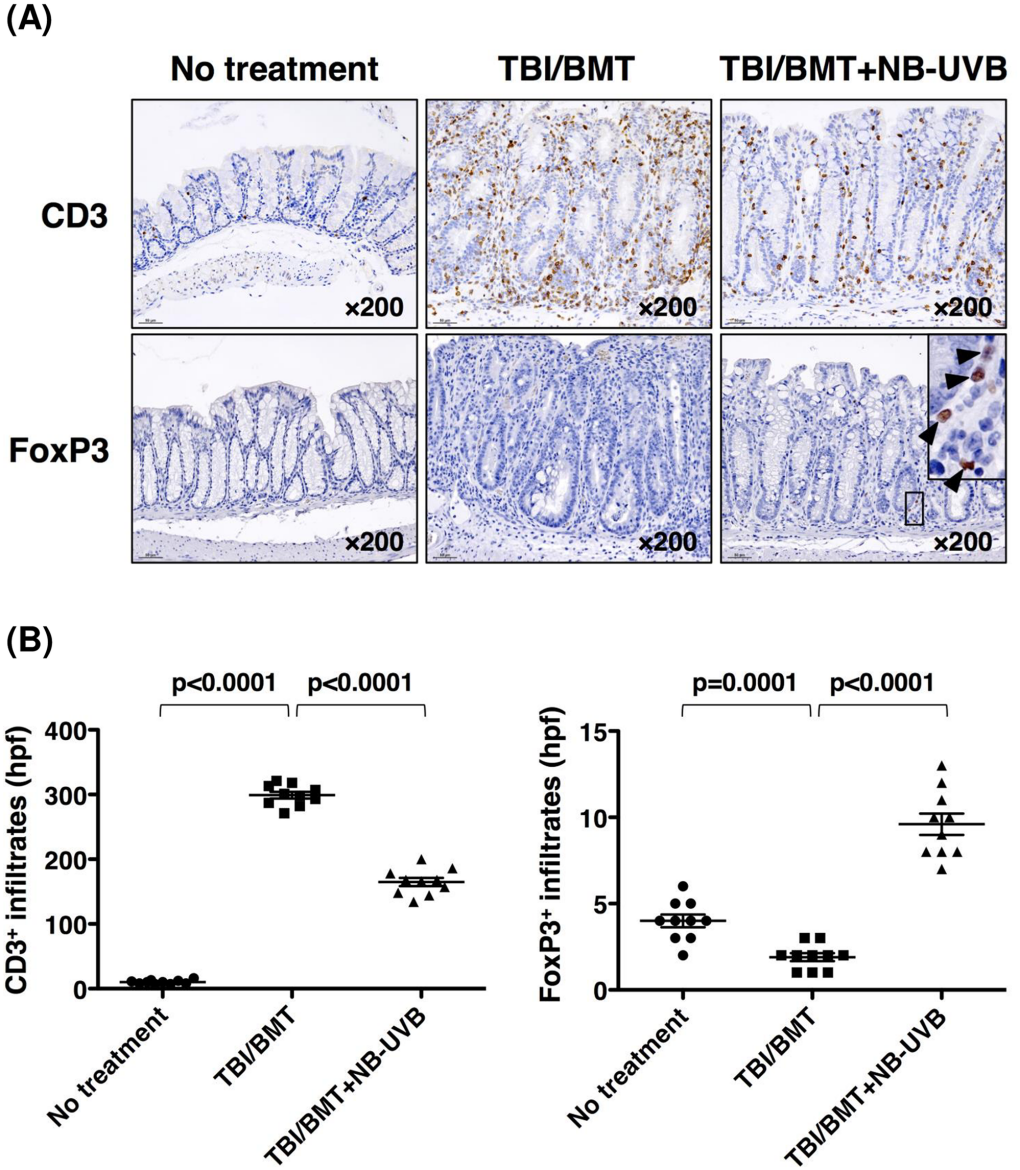
Effect of NB-UVB irradiation on Treg cells in intestine of aGVHD mice. Tissue samples were collected on day 35 after BMT. (A) Immunohistochemical staining of CD3 or FoxP3 was performed as described in the “Materials and Methods”. Photomicrographs are presented as a typical image in each group. Magnification is indicated in each photomicrograph. The filled triangles in the insert of the right lower panel show FoxP3^+^ cells. (B) CD3 or FoxP3-positive cells in each section were semi-quantitatively counted under ×400 magnification [high-power field (hpf)]. Ten non-overlapping views were obtained from each section. The data are representative of three separate experiments and presented as the mean±SEM.

Based on these data, we speculated that Treg cells are involved in the mechanism whereby NB-UVB irradiation ameliorated GVHD since Treg cells influenced immune pathways by secreting TGF-β and IL-10 to mediate direct inhibition of Th1, Th2 and Th17 cells [33]. Then, we examined the infiltration of FoxP3^+^ Treg cells into the intestine by immunohistochemical staining (Fig 5A), which was also evaluated by semi-quantitative analysis (Fig 5B). As control staining, CD3^+^ T lymphocytes were examined. Infiltration of CD3^+^ T lymphocytes in the intestine was massively induced in the “TBI/BMT” group compared with the “No treatment” group (p<0.0001). With NB-UVB irradiation, the infiltration of CD3^+^ T lymphocytes in the “TBI/BMT+NB-UVB” group was reduced compared with that in the “TBI/BMT” group (p<0.0001). In contrast, infiltration of FoxP3^+^ Treg cells in the intestine was reduced in the “TBI/BMT” group compared with the “No treatment” group (p = 0.001) and was enhanced upon stimulation of NB-UVB in the “TBI/BMT+ NB-UVB” group compared with the “TBI/BMT” group (p<0.0001). NB-UVB irradiation also increased the infiltration of FoxP3^+^ Treg cells in the lungs (S1 Fig) and skin (S2 Fig). The number of CD3^+^ T lymphocytes was slightly increased in the skin of the “TBI/BMT+NB-UVB” group, perhaps due to direct stimulation of NB-UVB (S2 Fig).

**Fig 6:**
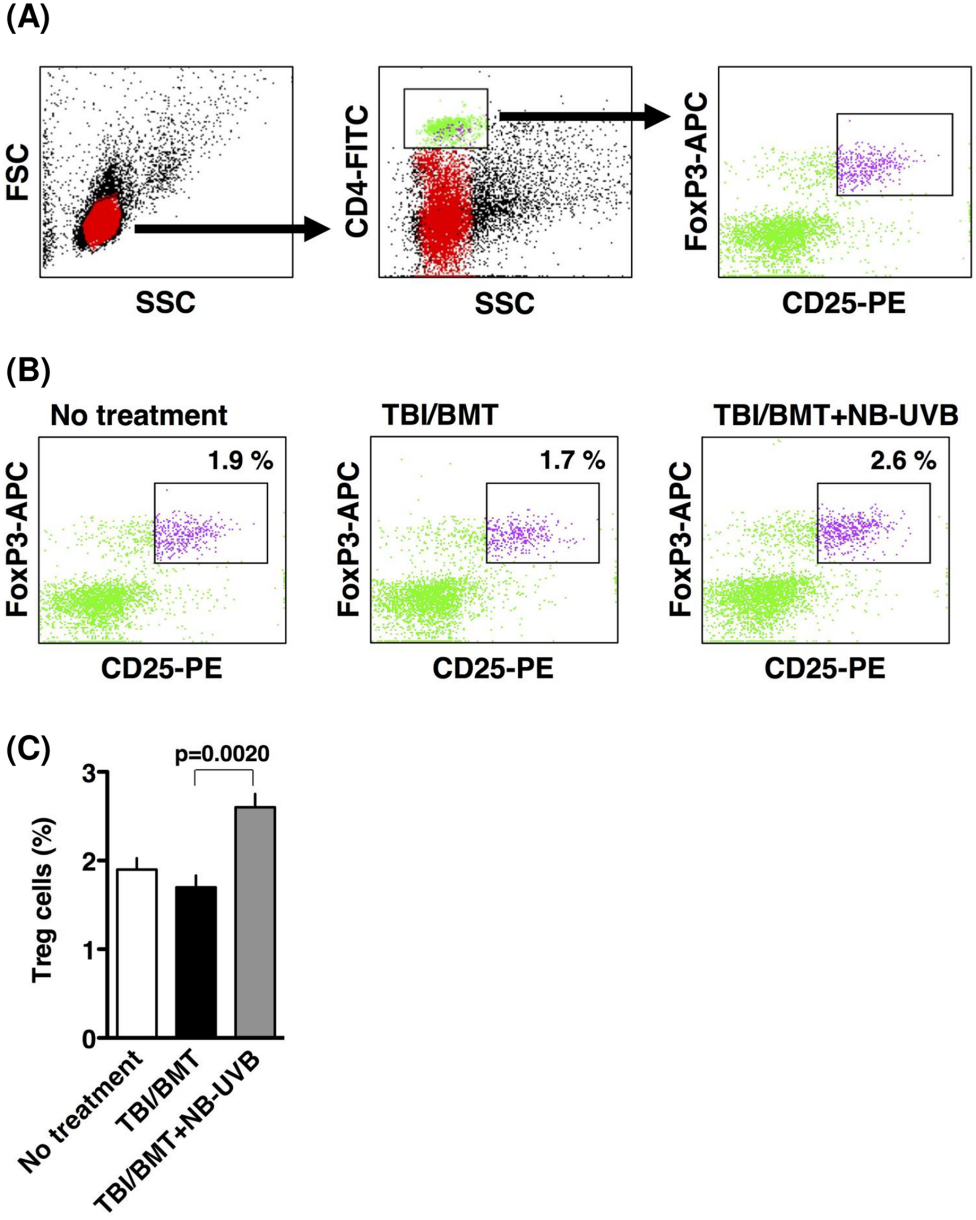
Effect of NB-UVB irradiation on Treg cells in spleen of aGVHD mice. Spleens were collected on day 35 after BMT. (A) Treg cells in spleen were detected by flow cytometry as CD4^+^CD25^+^FoxP3^+^ cells as described in the “Materials and Methods”. Gating was performed according to CD4 expression. (B) CD25/FoxP3 dot plots are presented as a typical image in each group. (C) Treg cells in spleen were counted by flow cytometry in each group. The data are representative of three separate experiments and presented as the mean±SEM.

To further confirm the results mentioned above, Treg cells in the spleen were analyzed. Treg cells were quantified as CD4^+^CD25^+^FoxP3^+^ cells using flow cytometry, in which CD4-negative cells were gated out from a group of lymphocytes, followed by counting CD25^+^FoxP3^+^ cells in the “No treatment”, “TBI/BMT”, or “TBI/BMT+NB-UVB” group (Fig 6A and 6B). As shown in Fig 6C, NB-UVB stimulation significantly increased the number of CD4^+^CD25^+^FoxP3^+^ Treg cells in the “TBI/BMT+NB-UVB” group compared with the “TBI/BMT” group (p = 0.0020).

## Discussion

NB-UVB with a peak at 311 nm, which is considered to be more effective than broadband UVB, has been widely used in dermatological phototherapy. In particular, it is a well-established treatment modality for psoriasis [34].

As for the application of NB-UVB phototherapy to GVHD, we and others have reported that it was highly efficacious for cutaneous lesions of both acute and chronic GVHD [11–16]. It was speculated that NB-UVB irradiation ameliorated skin GVHD by directly inducing apoptosis of inflammatory lymphocytes [17]; however, we clearly demonstrated in our previous report [11] that NB-UVB irradiation induces expansion of Treg cells in peripheral blood of skin aGVHD patients. If our previous findings were really so, NB-UVB phototherapy should be effective for not only skin GVHD but also GVHD of internal organs such as the intestine and liver.

To date, it has been uncertain whether the effect of NB-UVB reaches internal organs affected by GVHD. We, in this paper, clearly demonstrated that NB-UVB phototherapy dramatically rescued a murine lethal aGVHD model of the intestine. To the best of our knowledge, this is the first report demonstrating that NB-UVB has the ability to ameliorate GVHD of internal organs through the expansion of Treg cells.

On the other hand, another advancement in dermatological phototherapy is extracorporeal photopheresis (ECP) [35,36]. ECP is an autologous cell therapy that is widely used for the treatment of T cell-mediated diseases. The ECP treatment consists of the irradiation of ultraviolet A to peripheral blood mononuclear cells collected by apheresis in the presence of a photosensitizing agent (8-methoxypsoralen). This will lead to an irreversible DNA crosslink, culminating in apoptosis of the treated cells. Then, the treated cells are re-infused to the patient.

ECP has shown potent clinical benefits in patients with various T cell-mediated diseases including GVHD. It leads to a decrease or a complete withdrawal of immunosuppressive drugs, thus avoiding steroid-related side effects in GVHD patients [37]. Even for patients with steroid-refractory acute and chronic GVHD, ECP is an effective therapeutic modality [38].

Needless to say, both ECP and NB-UVB are promising secondary therapies for GVHD and we cannot discuss the superiority of one or the other treatment until phase III clinical trials are conducted and completed. However, NB-UVB therapy has some advantages: since ECP is an apheresis-based procedure, it is contraindicated in patients who cannot tolerate extracorporeal volume loss and those who have coagulation disorders such as thrombocytopenia and disseminated intravascular coagulation. NB-UVB therapy does not have such contraindications.

As for the mechanism whereby NB-UVB irradiation is efficacious for internal organs such as the intestine, we have clearly demonstrated the possible involvement of Treg cells on the basis of the findings of increased levels of serum Treg cytokines such as TGFβ and IL-10, the infiltration of FoxP3^+^ cells into inflamed tissue of intestine, and the increased number of CD4^+^CD25^+^FoxP3^+^ cells in spleen were evoked by NB-UVB phototherapy.

The reason why NB-UVB irradiation induces the expansion of Treg cells was suggested in previous papers [39,40]. Loser K et al. [39] reported that receptor activator of NF-κB ligand (RANKL) was upregulated in keratinocytes of inflamed skin irradiated by UVB, resulting in functional alterations of epidermal dendritic cells and systemic increases in Treg cells. In our experiments with NB-UVB irradiation, the same mechanism may be involved in the expansion of Treg cells.

As another plausible mechanism, we should take a link between UVB-skin and neuro-immune homeostasis into consideration [41–43]. Skin being the largest organ is actively engaged in the maintenance of homeostasis by production of neuropeptides, hormones and by generating neural signals, which may reach different organs [44–48]. We will investigate the regulation of local and global homeostasis by the skin neuroendocrine system in our next experiments.

Unexpectedly, we found that NB-UVB irradiation ameliorated IPS as well as aGVHD of the intestine in our experimental model (Fig 3). Treg cells were increased throughout the inflamed tissue of lungs of mice irradiated with NB-UVB (S1 Fig).

IPS is widespread alveolar injury in the absence of infection, cardiac dysfunction, renal failure, and iatrogenic fluid overload, and affects 5% to 25% of allo-HSCT recipients [29–31]. The mortality rate of IPS in allo-HSCT recipients ranges from 60 to 80% overall, and is greater than 95% among patients requiring mechanical ventilation [31].

Current standard treatment strategies for IPS include supportive care measures in conjunction with broad-spectrum antimicrobial agents and intravenous corticosteroids: however, responses to these standard therapies are limited [31]. To the best of our knowledge, there has been no report suggesting that the expansion of Treg cells could be an effective treatment for IPS. We expect that accumulating evidence will clarify the potential of Treg cells as a treatment strategy for IPS.

In conclusion, NB-UVB phototherapy was highly effective in a murine lethal aGVHD model of the intestine and complicating IPS. The mechanism whereby NB-UVB ameliorates GVHD of internal organs might be the induction of Treg cells. The results in this paper will be a rationale for our upcoming phase II study of NB-UVB phototherapy in aGVHD patients with involvement of intestine/liver or with complicating IPS.

## Supporting Information

S1 Fig Effect of NB-UVB irradiation on Treg cells in the lungs of aGVHD mice. The experimental design was the same as that described in the legend of Fig 5.
(PDF)

S2 Fig Effect of NB-UVB irradiation on Treg cells in skin of aGVHD mice. The experimental design was the same as that described in the legend of Fig 5.
(PDF)
