# Supplementary figures and images for: Narrow-Band Ultraviolet B Phototherapy Ameliorates Acute Graft-Versus-Host Disease of the Intestine by Expansion of Regulatory T Cells
Source: PLoS One. 2016 Mar 31;11(3):e0152823. doi: 10.1371/journal.pone.0152823 (PMC4816442; doi:10.1371/journal.pone.0152823)

S1 Fig

(A)

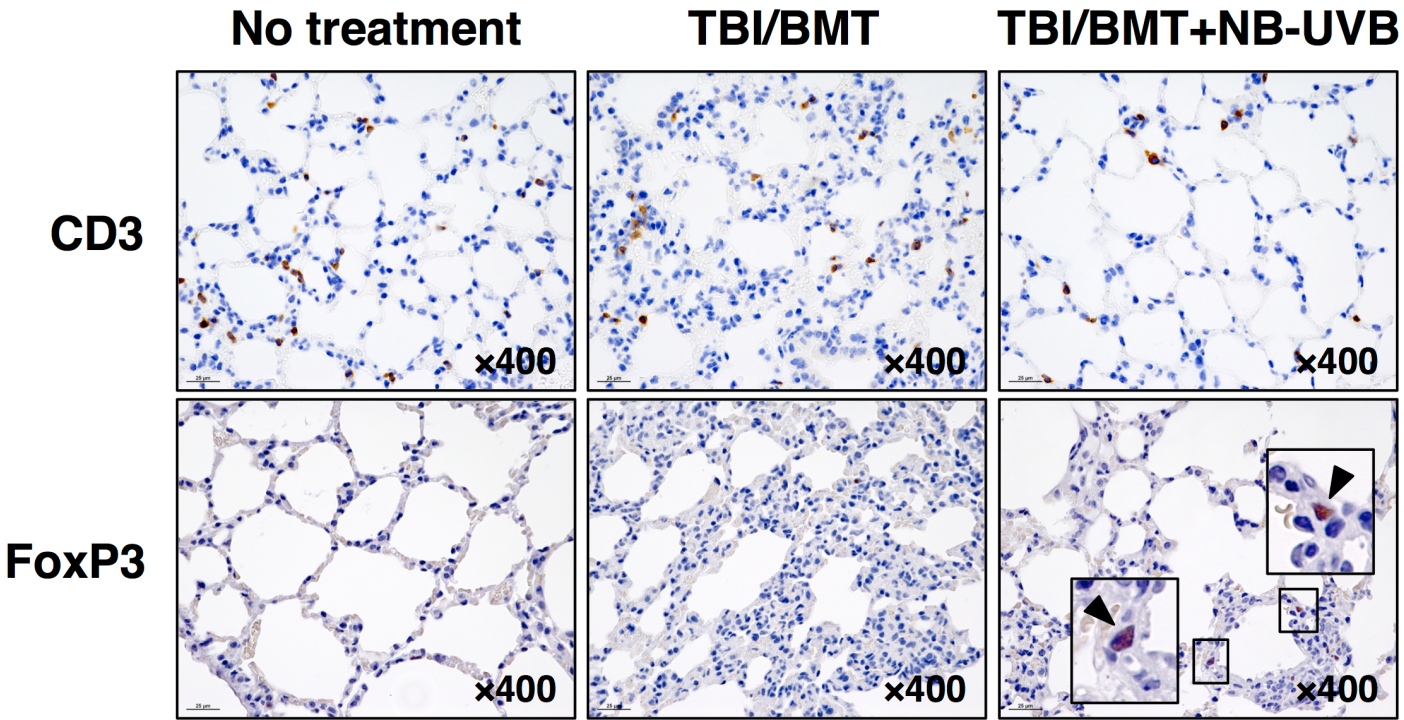

(B)

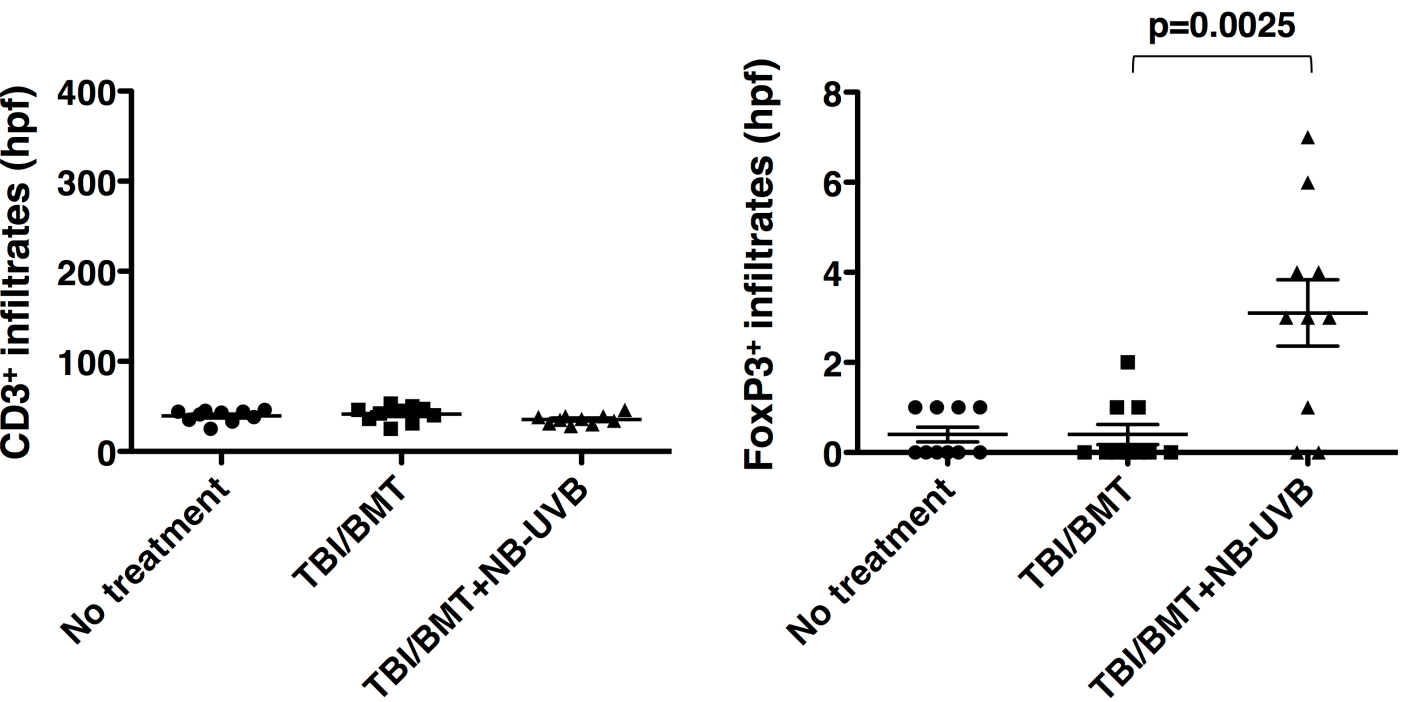

Supplement: S1 Fig — The experimental design was the same as that described in the legend of Fig 5. (PDF) [file pone.0152823.s001.pdf]

S2 Fig

(A)

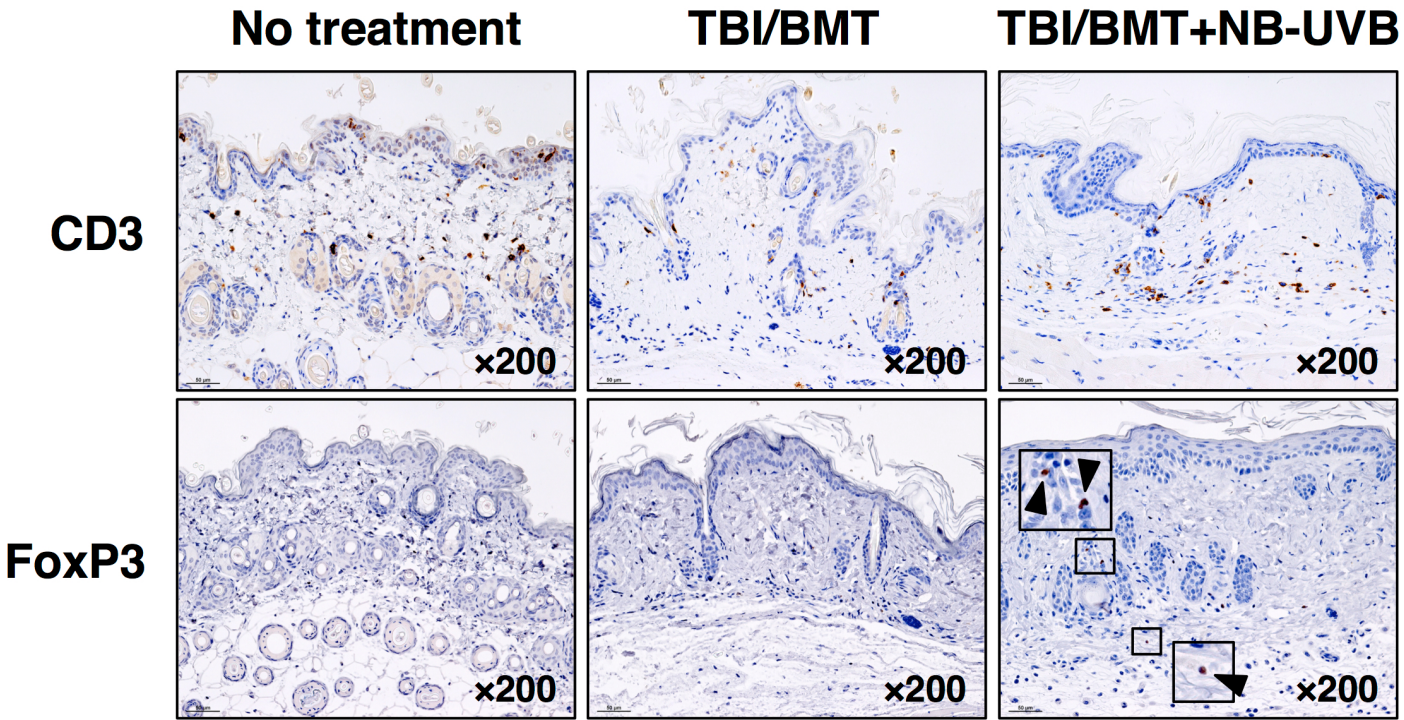

(B)

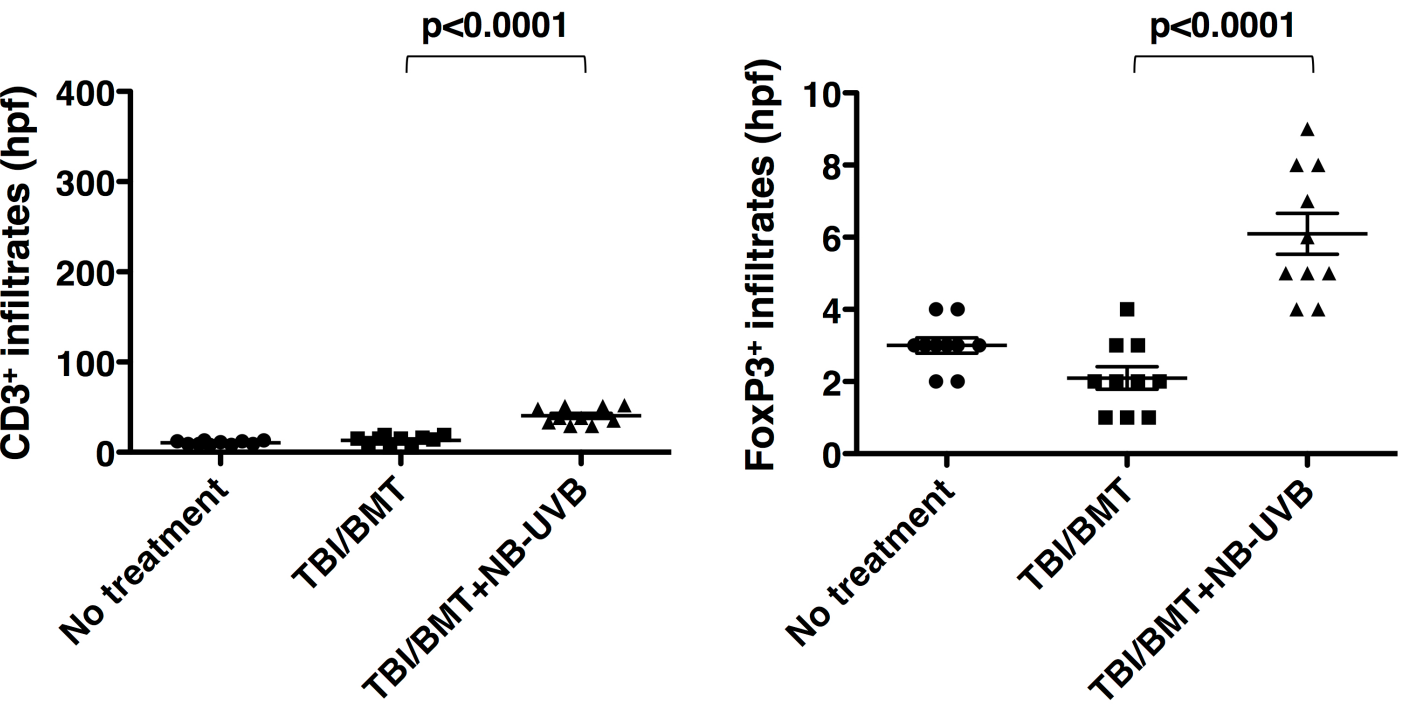

Supplement: S2 Fig — The experimental design was the same as that described in the legend of Fig 5. (PDF) [file pone.0152823.s002.pdf]
